# Impact of Delay to Cryopreservation on RNA Integrity and Genome-Wide Expression Profiles in Resected Tumor Samples

**DOI:** 10.1371/journal.pone.0079826

**Published:** 2013-11-20

**Authors:** Elodie Caboux, Maria Paciencia, Geoffroy Durand, Nivonirina Robinot, Magdalena B. Wozniak, Françoise Galateau-Salle, Graham Byrnes, Pierre Hainaut, Florence Le Calvez-Kelm

**Affiliations:** 1 Laboratory Services and Biobank, International Agency for Research on Cancer, Lyon, France; 2 Department of Pathology, Centre Hospitalier Universitaire de Caen, Caen, France; 3 Genetic Cancer Susceptibility Group, International Agency for Research on Cancer, Lyon, France; 4 Genetic Epidemiology Group, International Agency for Research on Cancer, Lyon, France; 5 Biostatistics Group, International Agency for Research on Cancer, Lyon, France; 6 International Agency for Research on Cancer, Lyon, France; 7 International Prevention Research Institute, Lyon, France; University of Connecticut Health Center, United States of America

## Abstract

The quality of tissue samples and extracted mRNA is a major source of variability in tumor transcriptome analysis using genome-wide expression microarrays. During and immediately after surgical tumor resection, tissues are exposed to metabolic, biochemical and physical stresses characterized as “warm ischemia”. Current practice advocates cryopreservation of biosamples within 30 minutes of resection, but this recommendation has not been systematically validated by measurements of mRNA decay over time. Using Illumina HumanHT-12 v3 Expression BeadChips, providing a genome-wide coverage of over 24,000 genes, we have analyzed gene expression variation in samples of 3 hepatocellular carcinomas (HCC) and 3 lung carcinomas (LC) cryopreserved at times up to 2 hours after resection. RNA Integrity Numbers (RIN) revealed no significant deterioration of mRNA up to 2 hours after resection. Genome-wide transcriptome analysis detected non-significant gene expression variations of −3.5%/hr (95% CI: −7.0%/hr to 0.1%/hr; p = 0.054). In LC, no consistent gene expression pattern was detected in relation with warm ischemia. In HCC, a signature of 6 up-regulated genes (*CYP2E1, IGLL1, CABYR, CLDN2, NQO1, SCL13A5*) and 6 down-regulated genes (*MT1G, MT1H, MT1E, MT1F, HABP2, SPINK1*) was identified (FDR <0.05). Overall, our observations support current recommendation of time to cryopreservation of up to 30 minutes and emphasize the need for identifying tissue-specific genes deregulated following resection to avoid misinterpreting expression changes induced by warm ischemia as pathologically significant changes.

## Introduction

Whole-genome expression profiling using microarrays has proven to be a powerful and reliable tool for classifying tumors and identifying predictors of therapeutic responses [1], [2]. Successful genome-wide expression profiling studies in the cancer genomics field include the characterization of subclasses of various cancers, such as leukaemia [3], breast carcinomas [4], [5], melanomas [6], lung [7] and hepatocellular carcinomas [8]. It was also shown for example that gene expression signatures in breast tumors were characteristic for *BRCA1* or *BRCA2* mutation carriers [9]. These new molecular markers are promising for diagnosis and prognostic improvement but also for prediction of response to therapy. Molecular profiling has been successfully used to evaluate and predict chemotherapy response [10], [11] and cancer survival outcome [12], [13].

While protocol standardization and reliable performance of genome-wide expression microarrays now generate high quality and consistent data across platforms [1], the accuracy of the array-based information can be affected by the quality of the biosample itself. Tumor tissue collection is a complex pre-analytical process due to the succession of steps from tissue resection to RNA extraction including freezing, cryopreservation, thawing and processing. During surgical tumor resection, tissues are exposed to multiple stresses such as decreased oxygen supply, temperature variations and mechanical and structural stress. This leads to a condition called *warm ischemia* that may significantly alter sample quality. RNA alterations and biological changes induced by those surgical stresses have been reported to bias transcriptomic analysis results as recently reviewed by Ma *et al*., who identified two different types of stress, warm ischemia-induced RNA degradation and warm ischemia-induced metabolic activity [14]. Moreover, Huang *et al*. have observed that gene expression may be altered up to 20-fold during 60 minutes of ischemia. These authors suggest that the majority of alterations that would be considered experimentally significant occur after 20 minutes of ischemic time [15]. Borgan *et al*. have also found that expression of specific miRNAs and mRNAs were significantly altered with ischemia time up to six hours after breast cancer surgery [16]. Thus, the notion of physical and biological quality of RNA from frozen specimens stored in tumor banks is becoming essential to insure high-quality specimen procurement and reliable comparisons between different transcriptomic studies. While the most frequent recommendation adopted by tumor banks is to freeze tissue specimens within minutes after surgical resection, there is little scientific evidence supporting this recommendation [17]. Identification of gene expression variations due to *ex vivo* warm ischemia occurring during tumor resection may provide an objective measure of mRNA decay during tissue processing and may also identify expression profiles of specific mRNA or mRNA families that could be used as markers of biological quality of tissue specimens.

Using two types of tumors, hepatocellular carcinoma (HCC) and non-small cell lung carcinomas (LC), we have investigated (i) whether delaying tissue snap-freezing after surgery has a significant impact on RNA integrity and genome-wide expression profiling analysis and if so, (ii) whether it is possible to establish a transcriptomic signature related to *ex vivo* warm ischemia that would help in avoiding misinterpretation of gene expression results.

## Results

### 1. RNA Sample and Microarray Quality

**Figure 1:**
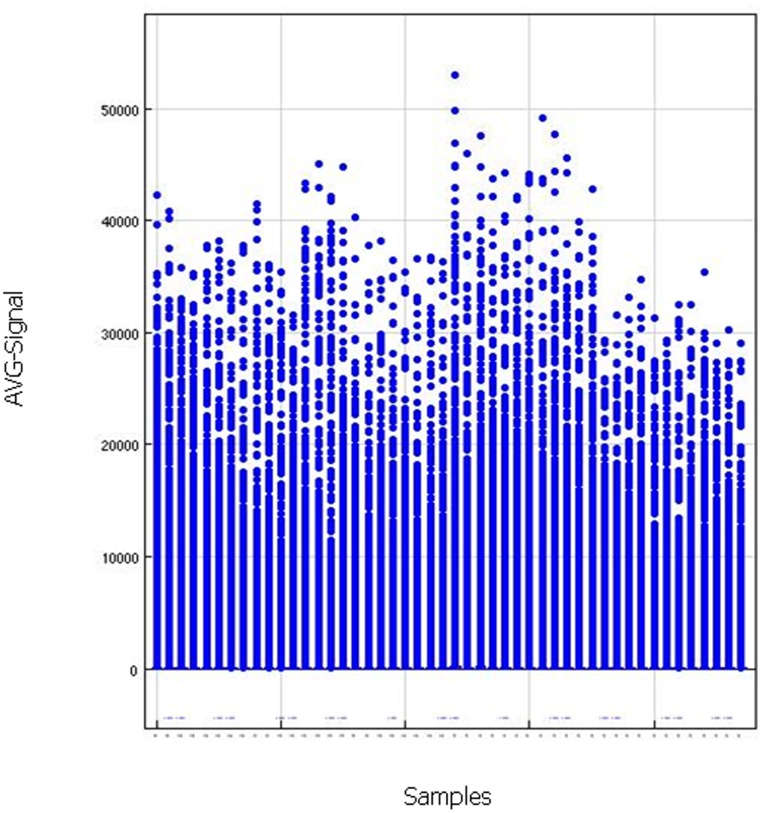
Within- and between-sample variance box-plot of microarray non-normalized fluorescent signals. The non-normalized fluorescent signals (AVG_Signal) have been generated by the Illumina Genome Studio V2010.2 for the 3 HepatoCellular Carcinomas (HCC) and the 3 Lung Carcinomas (LC ) samples taken at the center and at the periphery of the tumors and maintained at room temperature and then frozen in liquid nitrogen at different times: 5 minutes (t5, reference time), 15 minutes (t15), 30 minutes (t30) and 120 minutes (t120).

Table 1 shows RNA Integrity Numbers and microarray quality indicators for the various samples analyzed. Overall, HCC and LC combined, no deterioration of RNA Integrity Number (RIN) was observed with increasing time to cryopreservation (p = 0.38) but lower RIN values for LC were observed (p<0.001) (Table 1). There was no association of RIN numbers with tumor origin (peripheral versus central, p = 0.40). Size distribution of *in vitro* transcription products (cRNAs) was homogeneous across samples and Illumina array summary plot including hybridization, labelling, background and housekeeping genes controls showed satisfactory overall performance of the arrays (data not shown). The number of genes detected on the Illumina array was independent of time to cryopreservation (p = 0.80), RIN (p = 0.095) and central versus peripheral origin (p = 0.68), but was 3.7% higher for LC than HCC (p = 0.005). All samples but one passed the quality criterion P95/P05>10. The exception, LC6_15C, was borderline with P95/P05 = 9.51 (Table 1). Moreover, Figure 1 shows the distribution of the fluorescent signals across the arrays and the between array variance is not excessively large compared to that within each array. Overall, these results provide no indication that stress induced by 2 hours delay to cryopreservation impacted integrity of extracted RNA or microarray performance.

**Table 1:** Samples description and microarray quality.

Patient	Tumor pathology	Delay to cryopreservation (minutes)	Site	RIN Number	Detected Genes at p<0.01	p95/p05	Scatter plot r^2^
4	HCC	5	Central	8	9396	21.65	
4	HCC	5	Peripheral	7.4	9521	17.60	0.9763
4	HCC	15	Central	7.6	9175	21.22	
4	HCC	15	Peripheral	7.8	9486	20.77	0.9661
4	HCC	30	Central	7.9	9583	17.25	
4	HCC	30	Peripheral	7.8	9184	15.78	0.9824
4	HCC	120	Central	7.6	9171	13.83	
4	HCC	120	Peripheral	7.3	9319	11.88	0.9803
5	HCC	5	Central	7.2	10154	13.45	
5	HCC	5	Peripheral	6.7	9841	12.76	0.9749
5	HCC	15	Central	7.1	9627	11.42	
5	HCC	15	Peripheral	7.7	10120	16.52	0.8487
5	HCC	30	Central	7.6	10178	15.69	
5	HCC	30	Peripheral	7.3	10072	14.03	0.9792
5	HCC	120	Central	7.8	9677	11.75	
5	HCC	120	Peripheral	7.1	10250	17.37	0.874
6	HCC	5	Central	6.9	10994	17.49	
6	HCC	5	Peripheral	6.8	10437	16.32	0.9795
6	HCC	15	Central	7	10407	16.88	
6	HCC	15	Peripheral	6.9	10750	16.41	0.9604
6	HCC	30	Central	6.6	10732	16.13	
6	HCC	30	Peripheral	7.1	10262	15.93	0.9801
6	HCC	120	Central	6.9	10392	17.12	
6	HCC	120	Peripheral	6.5	10393	16.70	0.8507
2	LC	5	Central	6.7	11775	23.07	
2	LC	5	Peripheral	5.4	11576	17.89	0.9599
2	LC	15	Central	6	11952	24.63	
2	LC	15	Peripheral	5.2	11551	18.41	0.9573
2	LC	30	Central	6.8	11497	19.48	
2	LC	30	Peripheral	7.5	12078	20.67	0.9697
2	LC	120	Central	6.2	12036	21.07	
2	LC	120	Peripheral	6.2	12300	16.84	0.9738
5	LC	5	Central	6	11591	21.70	
5	LC	5	Peripheral	6.2	11061	24.18	0.9593
5	LC	15	Central	6.1	11004	21.78	
5	LC	15	Peripheral	6	11150	24.02	0.9832
5	LC	30	Central	5.7	10054	14.88	
5	LC	30	Peripheral	6.2	10696	15.49	0.9644
5	LC	120	Central	6.6	11078	17.06	
5	LC	120	Peripheral	6.2	11144	19.20	0.9801
6	LC	5	Central	6.9	10521	14.18	
6	LC	5	Peripheral	6.8	10413	17.40	0.9652
6	LC	15	Central	ND	10519	9.51	
6	LC	15	Peripheral	5.8	10556	15.29	0.9631
6	LC	30	Central	6.6	10511	19.14	
6	LC	30	Peripheral	5.9	10688	17.85	0.9791
6	LC	120	Central	6.4	11090	17.74	
6	LC	120	Peripheral	7.8	10095	10.39	0.9603

Type of tumor and delay to tumor freezing are shown. RNA integrity is evaluated through the RIN number. The ratio of centiles P95/P05 reflects the overall strength of the signal compared to the background. The Pearson correlation coefficient (r^2^) shows the correlation between log-expression levels of the central and peripheral samples, for each tumor and each time to cryopreservation.

HCC: HepatoCellular Carcinoma.

LC: Lung Carcinoma.

ND: Not Determined.

### 2. Overall Gene Expression

There was mild evidence for a slow decline in expression level by 3.5%/hr (95% CI −7%/hr to 0.1%/hr; p = 0.054) in relation with delay to cryopreservation (Table 2). There was no indication of an overall quadratic dependence on time (p = 0.27), nor was there any effect when analyzing the tumor sites separately or when analyzing peripheral versus central tumor origin (Table 2). Moreover, the correlation coefficient r^2^ (Table 1) is close to 1 for each comparison, suggesting that central and peripheral specimens could be considered as technical replicates in time course analyses. Restricting the analysis to genes in the highest or lowest 5% of geometric mean expression across all time points for combined HCC and LC did not change the results (Table 2). Interestingly, the genes in the lowest 5% of the geometric mean expression distribution showed a significant consistent decline in LC samples (−2.5%/hr, 95% CI −4.3%/hr to −0.8%/hr; p = 0.004). It remains possible that this is a chance finding due to the numerous sub-set analyses.

**Table 2:** Gene expression for all probes and for restricted sets of probes.

		Rate of change (%/hr)	95%CI	p value
All probes				
All samples		−3.5	−7.0 to 0.1	0,054
HCC		−2.3	−7 to 2.3	0,33
LC		−4.6	−9.8 to 0.6	0,09
Central tumor		−3.5	−8.6 to 1.6	0,09
Peripheral tumor		−3.4	−8.2 to 1.4	0,17
Probes with the lowest 5% of geometric mean expression				
All samples		−1.7	−3 to 0.4	0,009
HCC		−0.8	−2.6 to 0.9	0,35
LC		−2.5	−4.3 to −0.8	0,004
Probes with the highest 5% of geometric mean expression				
All samples		−8	−16.2 to 0.1	0,054
HCC		−5.9	−15 to 3.8	0,23
LC		−10.2	−23 to 2.9	0,13
Probes in warm ischemia genes				
All samples		−4.7	13 to 3.6	0,27
HCC		7.3	−18 to 2.9	0,16
LC		2.2	−16 to 11	0,75
Probes in HCC genes				
HCC		−8,5	−24 to 7.2	0,29

Over-all rate of expression changes for all probes in all samples combined, in LC and HCC samples and in peripheral and central samples are estimated as percent-change per hour. Expression levels changes are also estimated for different sets of probes (lowest and highest 5% of geometric mean expression, probes in warm ischemia genes and probes in HCC genes).

### 3. Analysis of Individual Genes

**Figure 2:**
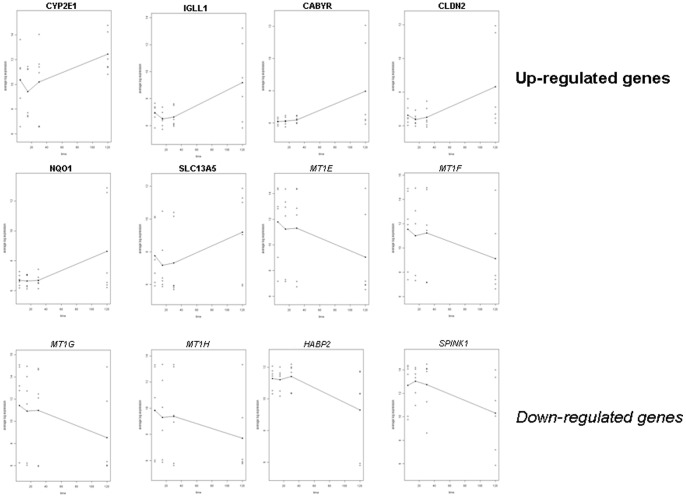
Average log-expression profiles of the 12 genes with significant up- or down-regulation over harvesting time (FDR<0.05) in HCC. The BRB-ArrayTools v4.2 time course analysis model was applied to whole-genome expression microarray data (HCC and LC samples) to identify significant individual deregulated genes over harvesting time. No significant deregulated genes in LC were observed. Individual log-expression profiles (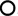) and average log-expression line plots (3 HCC samples taken at the center and at the periphery) in relation to delay to tumor cryopreservation are displayed.

A total of 9,191 of 48,783 probes (18.8%) passed the probe filtering criteria and were hence eligible for testing for changes in expression. At the 5% FDR level, the ANOVA model identified 12 genes in HCC whose expression varied with delay to cryopreservation (6 up-regulated: *CYP2E1, IGLL1, CABYR, CLDN2, NQO1, SCL13A5* and 6 down-regulated: *MT1G, MT1H, MT1E, MT1F, HABP2, SPINK1*), whereas no gene expression variation in LC was observed. The list of genes associated with time since resection, with their corresponding expression profiles is given in Figure 2.

### 4. Exploratory Cluster Analysis

**Figure 3:**
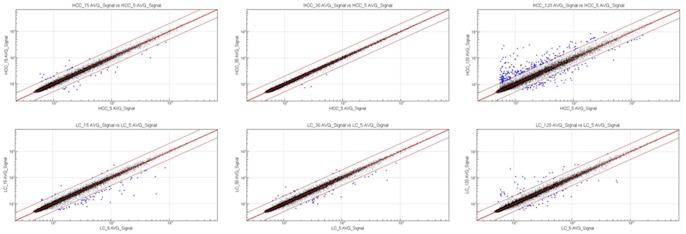
Hierarchical cluster analysis of all samples following log-transformation and quantile normalization of the microarray data. Dendrogram for clustering experiments was created using centred correlation and average linkage method. Length of nodes corresponds to correlation between samples. HCC4_5P: HCC from patient 4 taken at the periphery of the tumor and maintained at room temperature and then frozen in liquid nitrogen at t5 (min).

**Figure 4:**
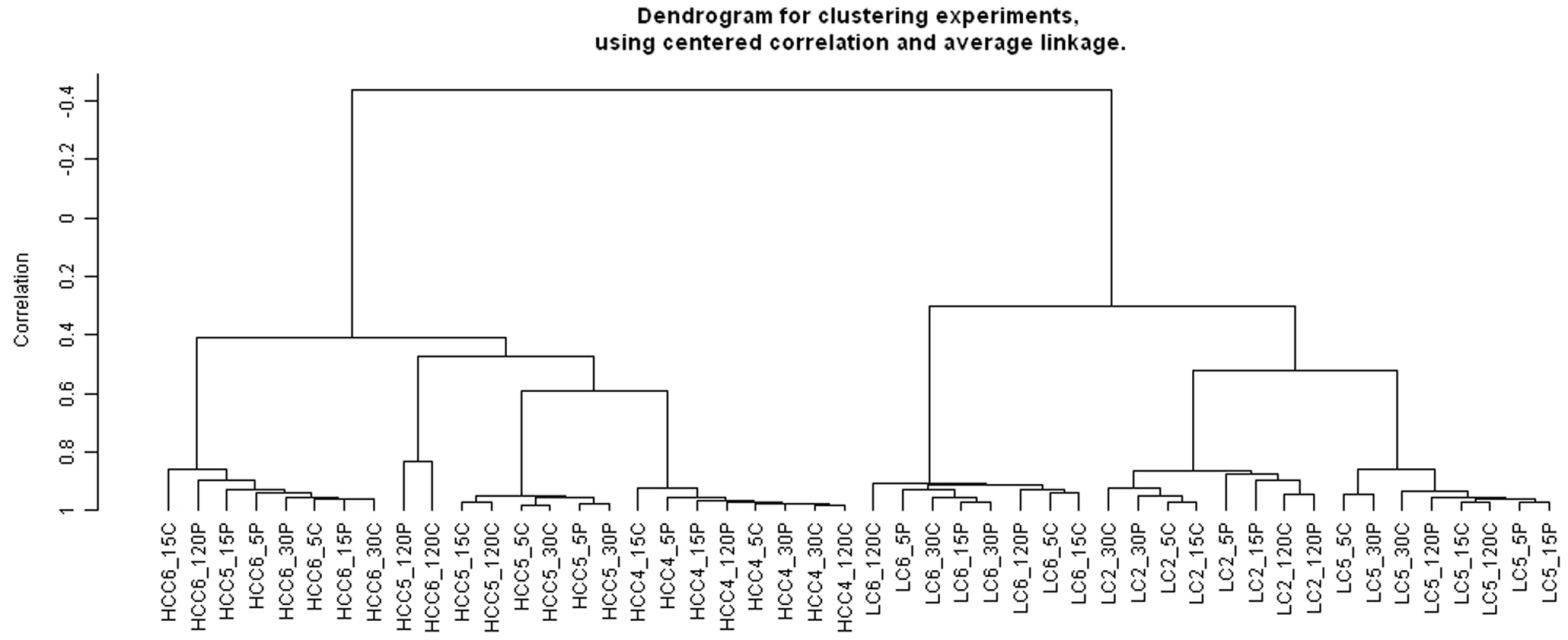
Time-course scatter-plots of HCC and LC genome-wide expression profiling quantile normalized data. Scatter plots for each tumor pair at t5 (HCC_5_AVG_Signal and LC_5_AVG_Signal on the X Axis) versus harvested tumor pairs at t15, t30 and t120 (on the Y Axis) were generated on a logarithmic scale. Genes showing greater than 2-fold change relative to the t5 sample from the same tumor were highlighted.

Figure 3 shows the dendrogram obtained from an unsupervised clustering of all samples analyzed. This dendrogram shows a first hierarchy of clusters by tumor types (HCC and LC) with subsequent clustering of tumors from each patient. While all experimental conditions from the same LC patients clustered together, the samples of HCC5 preserved at 15 min and 120 min and of HCC6 preserved at 120 min did not cluster together (Figure 3). It is important to note that, for these two patients, the second samples (peripheral or central) for those preservation times clustered well within the same patient. However, within each cluster of tumor samples, we did not observe any correlation with increased delay to cryopreservation. This suggests that if there was a change in expression with time, this change did not proceed according to a systematic pattern. This view was further supported by differential expression analysis performed with Illumina Genome Studio V2010.2 software. Scatter plots (Figure 4) showed that for each tumor type the correlation between log-expression at time t5 and later was close to 1. Thus, there was little evidence for large-scale re-ordering of expression level over time, despite a tendency for an increased number of outliers at t120, in particular in HCC.

### 5. Restricted Gene Expression Analysis to Ischemic Genes and HCC-specific Genes

While the expression of several genes have been reported to be consistently deregulated at the early stage after surgery, the time-course analysis of individuals genes performed with our sample series did not reveal any of those genes. Therefore, we analyzed independently 21 genes in our dataset (*JNK3, JUNB, AP1B1, AP1S1, AP1M1, AP1S1, CA-IX, HHR6B, PRSS25, FOS, HIF1A, HO-1, JUND, JUN, KRT19, KRT20, CEA, KLF6, MDM4, FBLN2, FGRF4)* that have been reported as deregulated at early stage after surgery [14], [19], [20]. Average rates of expression change for those 21 genes showed no evidence for a gene expression deregulation over time delay to tumor freezing (Table 2).

In addition, taking advantage of the publicly available Liverome database, which provides a large collection of well-curated HCC gene expression signatures (http://liverome.kobic.re.kr), we selected the 34 most biologically relevant HCC genes, reported as deregulated in more than 4 studies recorded in the Liverome database [23], [24], [25], [26], [27], [28], [29], [30], [31], [32], [33], [34], [35], [36], [37], [38], [39], [40], [41], [42], [43]. We then conducted analysis of the rates of expression changes to the 34 HCC-specific genes in our HCC dataset in order to examine whether some reported biologically significant HCC genes could be deregulated under warm ischemia conditions associated with delay to tumor freezing. Overall rates of expression changes for those 34 HCC genes showed no evidence for a gene expression deregulation over time delay to HCC tumor freezing (Table 2).

The Neyman’s test revealed that the rates of gene expression changes for the 21 selected ischemic genes and 34 HCC specific genes tended to the extremes of the distribution among the full panel of genes. Both the log-linear trends and the quadratic terms were significantly extreme (Ischemia genes: Linear effect beta = 0.94, SE = 0.18, p = 3.0E-7; Quadratic effect beta = 0.66, SE = 0.18, p = 3.0E-4; HCC genes: Linear effect beta = 1.16, SE = 0.14, p = 1.9E-16; Quadratic effect beta = 1.35, SE = 0.14, p = 1.9E-21). However, strikingly, after normalizing the estimated parameters by their standard errors, the re-calculated ranks were completely consistent with a uniform distribution among all genes (Ischemia genes: Linear effect beta = 0.12, SE = 0.18, p = 0.53, Quadratic effect beta = −0.26, SE = 0.18, p = 0.16; HCC genes: Linear effect beta = 0.13, SE = 0.14, p = 0.36, Quadratic effect beta = −0.07, SE = 0.14, p = 0.60), suggesting that those genes could be systematically be prone to highly variable gene expression changes between and within samples.

Table 3 compares the individual expression changes reported in the Liverome database for the 34-HCC specific genes observed in more than 4 studies to the expression changes (%/hr) related to warm ischemia in our experimental HCC dataset. Overall, the comparison of the rate of expression changes observed in our HCC dataset for those 34 genes to the predicted behavior of the same genes in the Liverome studies revealed borderline significance (p = 0.057). While this difference becomes significant when adjusting for the quadratic effect (p = 0.032), the normalization of rate of expression changes by their standard errors abrogated the significance (p = 0.241), even after adjustment for the quadratic effect (p = 0.213). Of 34 genes reported as biologically significant in hepatocarcinogenesis, 16 (47%) were deregulated in the same direction (i.e., up-regulated in the Liverome database and in our dataset or vice versa), 12 (35%) of which showed a deregulation of more than 10%/hr over delay to cryopreservation and one of those 12 genes (*MT1F*) has also been identified as significantly down-regulated under warm ischemia conditions in our HCC dataset. Altogether, we cannot exclude that the deregulation of those 12 genes is not only associated with the hepatocarcinogenesis per se but also with ischemic stress that could differ within tumor sample series of studies reported in the Liverome database.

**Table 3:** List of 34 HCC specific genes: comparison of gene expression data from the Liverome database and experimental dataset.

			Liverome dataset				Experimental dataset		Conclusions
				Reported results					
Gene symbol	Entrez Gene ID	Gene name	Frequency(studies)	Up-regulated expression	Down-regulated expression	Expression reported with a p-value	Rate of expression change (%/h)	Up or down expression	Consistant results between datasets
A2M	2	alpha-2-macroglobulin	5		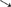 (5, 7, 11, 18)	<0.005 (9)	−0,062217	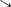	Yes
ADH4	127	alcohol dehydrogenase 4 (class II), pi polypeptide	4		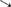 (17, 18, 19)	<0.005 (9)	0.0236812^a^	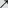	No
ALB	213	albumin	5^b^	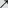 (20)	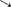 (6, 11, 18)	<0.005 (9)	0.04337605^a^	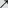	No
APOA1	335	apolipoprotein A-I	4^b^	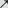 (20)	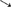 (11, 12)	<0.005 (9)	−0,2482746	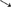	Yes
BHMT	635	betaine–homocysteine S-methyltransferase	5		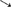 (5, 8, 12, 19)	<0.005 (9)	−0,3139206	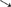	Yes
COL1A2	1278	collagen, type I, alpha 2	4	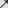 (7, 15, 17)		<0.005 (9)	−0.2465753^a^	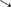	No
CYP3A4	1576	cytochrome P450, family 3, subfamily A, polypeptide 4	4^b^	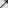 (20)	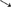 (1, 11)	<0.005 (9)	0,4847241	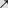	No
DUSP1	1843	dual specificity phosphatase 1	5^b^	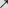 (11, 20)	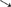 (14, 19)	<0.005 (9)	−0,1140679	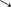	No
ECHS1	1892	enoyl CoA hydratase, short chain, 1, mitochondrial	6^b^	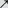 (21)	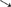 (1, 11, 14, 18)	<0.005 (9)	0,2454375	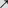	No
FAM36A	116228	family with sequence similarity 36, member A	4^b^	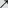 (20)	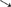 (11, 21)	<0.005 (9)	0,267976	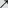	No
FGB	2244	fibrinogen beta chain	4		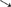 (11, 17, 18)	<0.005 (9)	−0.1665138^a^	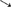	Yes
FGG	2266	fibrinogen gamma chain	4		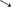 (1, 11, 21)	<0.005 (9)	−0.015657767^a^	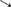	Yes
FN1	2335	fibronectin 1	4	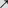 (5, 13, 16)		<0.005 (9)	−0.0767727^a^	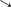	No
GMFG	9535	glia maturation factor, gamma	4	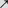 (11, 18, 19)		<0.005 (9)	0,100325	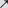	Yes
GPC3	2719	glypican 3	7	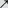 (4, 6, 10, 15, 17, 20)		<0.005 (9)	0,3639491	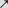	Yes
HAMP	57817	hepcidin antimicrobial peptide	4^b^	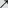 (20)	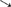 (6, 11)	<0.005 (9)	0,7744631	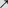	No
HGFAC	3083	HGF activator	4		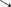 (5, 14, 17, 18)		0,5554259	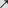	No
HPD	3242	4-hydroxyphenylpyruvate dioxygenase	4		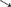 (6, 17, 18, 19)		0,4381038	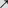	No
HSD17B6	8630	hydroxysteroid (17-beta) dehydrogenase 6 homolog (mouse)	4		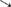 (1, 10, 18)	<0.005 (9)	−0,3196788	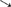	Yes
IGFBP3	3486	insulin-like growth factor binding protein 3	4		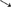 (2, 5, 11)	<0.005 (9)	−0.4585644^a^	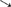	Yes
LCN2	3934	lipocalin 2	4	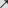 (3, 5, 15)		<0.005 (9)	0,279305	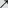	Yes
MT1F	4494	metallothionein 1F	4^b^	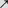 (20)	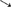 (8, 10, 17)		−0,4286072	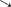	Yes
MT2A	4502	metallothionein 2A	5^b^	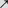 (20)	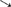 (6, 8, 18)	<0.005 (9)	−1,311318	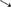	Yes
MT3	4504	metallothionein 3	4		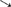 (5, 7, 8, 19)		−0,0167001	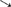	Yes
PCK1	5105	phosphoenolpyruvate carboxykinase 1 (soluble)	4		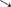 (8, 12, 18, 19)		0.0363959^a^	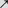	No
PLG	5340	plasminogen	5		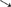 (5, 7, 10, 16)	<0.005 (9)	0,0354232	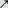	No
PRPSAP1	5635	phosphoribosyl pyrophosphate synthetase-associated protein 1	4	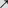 (11, 15, 19)		<0.005 (9)	−0,0122151	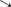	No
RHOB	388	ras homolog gene family, member B	4^b^	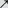 (16)	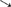 (5, 7)	<0.005 (9)	0,0256628	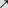	No
SAA2	6289	serum amyloid A2	4		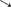 (1, 6, 19)	<0.005 (9)	0,0021512	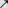	No
SLC22A1	6580	solute carrier family 22 (organic cation transporter), member 1	6^b^	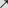 (20)	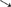 (6, 10, 11, 14)	<0.005 (9)	−0.21260725^a^	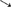	Yes
SPARC	6678	secreted protein, acidic, cysteine-rich (osteonectin)	7	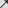 (3, 5, 7, 12, 15, 19)		<0.005 (9)	−0,1714643	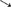	No
TDO2	6999	tryptophan 2,3-dioxygenase	5		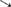 (1, 8, 11, 19)	<0.005 (9)	−0.0018556^a^	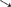	Yes
TUBA1B	10376	tubulin, alpha 1b	4	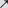 (2, 4, 5, 8)			−0,0030352	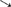	No
UBD	10537	ubiquitin D	7	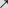 (4, 10, 12, 15, 17, 20)		<0.005 (9)	0,5806394	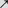	Yes

Expression trend of 34 HCC specific genes reported as deregulated in more than 4 studies in the public Liverome database was compared to experimental expression trend.

a average rate of expression from different Illumina probes.

b discrepancies between Liverome studies results.

Table 3 References.

1) Chan, K.Y., Lai, P.B., Squire, J.A., Beheshti, B., Wong, N.L., Sy, S.M., Wong, N., 2006. Positional expression profiling indicates candidate genes in deletion hotspots of hepatocellular carcinoma. Mod Pathol 19, 1546–1554.

2) Chung, E.J., Sung, Y.K., Farooq, M., Kim, Y., Im, S., Tak, W.Y., Hwang, Y.J., Kim, Y.I., Han, H.S., Kim, J.C., Kim, M.K., 2002. Gene expression profile analysis in human hepatocellular carcinoma by cDNA microarray. Mol Cells 14, 382–387.

3) Cui, X.D., Lee, M.J., Yu, G.R., Kim, I.H., Yu, H.C., Song, E.Y., Kim, D.G., 2010. EFNA1 ligand and its receptor EphA2: potential biomarkers for hepatocellular carcinoma. Int J Cancer 126, 940–949.

4) De Giorgi, V., Monaco, A., Worchech, A., Tornesello, M., Izzo, F., Buonaguro, L., Marincola, F.M., Wang, E., Buonaguro, F.M., 2009. Gene profiling, biomarkers and pathways characterizing HCV-related hepatocellular carcinoma. J Transl Med 7, 85.

5) Delpuech, O., Trabut, J.B., Carnot, F., Feuillard, J., Brechot, C., Kremsdorf, D., 2002. Identification, using cDNA macroarray analysis, of distinct gene expression profiles associated with pathological and virological features of hepatocellular carcinoma. Oncogene 21, 2926–2937.

6) Dong, H., Ge, X., Shen, Y., Chen, L., Kong, Y., Zhang, H., Man, X., Tang, L., Yuan, H., Wang, H., Zhao, G., Jin, W., 2009. Gene expression profile analysis of human hepatocellular carcinoma using SAGE and LongSAGE. BMC Med Genomics 2, 5.

7) Goldenberg, D., Ayesh, S., Schneider, T., Pappo, O., Jurim, O., Eid, A., Fellig, Y., Dadon, T., Ariel, I., de Groot, N., Hochberg, A., Galun, E., 2002. Analysis of differentially expressed genes in hepatocellular carcinoma using cDNA arrays. Mol Carcinog 33, 113–124.

8) Iizuka, N., Tsunedomi, R., Tamesa, T., Okada, T., Sakamoto, K., Hamaguchi, T., Yamada-Okabe, H., Miyamoto, T., Uchimura, S., Hamamoto, Y., Oka, M., 2006. Involvement of c-myc-regulated genes in hepatocellular carcinoma related to genotype-C hepatitis B virus. J Cancer Res Clin Oncol 132, 473–481.

9) Kato, K., Yamashita, R., Matoba, R., Monden, M., Noguchi, S., Takagi, T., Nakai, K., 2005. Cancer gene expression database (CGED): a database for gene expression profiling with accompanying clinical information of human cancer tissues. Nucleic Acids Res 33, D533–536.

10) Kim, B.Y., Lee, J.G., Park, S., Ahn, J.Y., Ju, Y.J., Chung, J.H., Han, C.J., Jeong, S.H., Yeom, Y.I., Kim, S., Lee, Y.S., Kim, C.M., Eom, E.M., Lee, D.H., Choi, K.Y., Cho, M.H., Suh, K.S., Choi, D.W., Lee, K.H., 2004. Feature genes of hepatitis B virus-positive hepatocellular carcinoma, established by its molecular discrimination approach using prediction analysis of microarray. Biochim Biophys Acta 1739, 50–61.

11) Kurokawa, Y., Matoba, R., Takemasa, I., Nakamori, S., Tsujie, M., Nagano, H., Dono, K., Umeshita, K., Sakon, M., Ueno, N., Kita, H., Oba, S., Ishii, S., Kato, K., Monden, M., 2003. Molecular features of non-B, non-C hepatocellular carcinoma: a PCR-array gene expression profiling study. J Hepatol 39, 1004–1012.

12) Lee, M.J., Yu, G.R., Park, S.H., Cho, B.H., Ahn, J.S., Park, H.J., Song, E.Y., Kim, D.G., 2008. Identification of cystatin B as a potential serum marker in hepatocellular carcinoma. Clin Cancer Res 14, 1080–1089.

13) Li, Y., Tang, R., Xu, H., Qiu, M., Chen, Q., Chen, J., Fu, Z., Ying, K., Xie, Y., Mao, Y., 2002. Discovery and analysis of hepatocellular carcinoma genes using cDNA microarrays. J Cancer Res Clin Oncol 128, 369–379.

14) Okabe, H., Satoh, S., Kato, T., Kitahara, O., Yanagawa, R., Yamaoka, Y., Tsunoda, T., Furukawa, Y., Nakamura, Y., 2001. Genome-wide analysis of gene expression in human hepatocellular carcinomas using cDNA microarray: identification of genes involved in viral carcinogenesis and tumor progression. Cancer Res 61, 2129–2137.

15) Patil, M.A., Chua, M.S., Pan, K.H., Lin, R., Lih, C.J., Cheung, S.T., Ho, C., Li, R., Fan, S.T., Cohen, S.N., Chen, X., So, S., 2005. An integrated data analysis approach to characterize genes highly expressed in hepatocellular carcinoma. Oncogene 24, 3737–3747.

16) Shirota, Y., Kaneko, S., Honda, M., Kawai, H.F., Kobayashi, K., 2001. Identification of differentially expressed genes in hepatocellular carcinoma with cDNA microarrays. Hepatology 33, 832–840.

17) Tackels-Horne, D., Goodman, M.D., Williams, A.J., Wilson, D.J., Eskandari, T., Vogt, L.M., Boland, J.F., Scherf, U., Vockley, J.G., 2001. Identification of differentially expressed genes in hepatocellular carcinoma and metastatic liver tumors by oligonucleotide expression profiling. Cancer 92, 395–405.

18) Xu, L., Hui, L., Wang, S., Gong, J., Jin, Y., Wang, Y., Ji, Y., Wu, X., Han, Z., Hu, G., 2001a. Expression profiling suggested a regulatory role of liver-enriched transcription factors in human hepatocellular carcinoma. Cancer Res 61, 3176–3181.

19) Xu, X.R., Huang, J., Xu, Z.G., Qian, B.Z., Zhu, Z.D., Yan, Q., Cai, T., Zhang, X., Xiao, H.S., Qu, J., Liu, F., Huang, Q.H., Cheng, Z.H., Li, N.G., Du, J.J., Hu, W., Shen, K.T., Lu, G., Fu, G., Zhong, M., Xu, S.H., Gu, W.Y., Huang, W., Zhao, X.T., Hu, G.X., Gu, J.R., Chen, Z., Han, Z.G., 2001b. Insight into hepatocellular carcinogenesis at transcriptome level by comparing gene expression profiles of hepatocellular carcinoma with those of corresponding noncancerous liver. Proc Natl Acad Sci U S A 98, 15089–15094.

20) Yamashita, T., Kaneko, S., Hashimoto, S., Sato, T., Nagai, S., Toyoda, N., Suzuki, T., Kobayashi, K., Matsushima, K., 2001. Serial analysis of gene expression in chronic hepatitis C and hepatocellular carcinoma. Biochem Biophys Res Commun 282, 647–654.

21) Zekri, A.R., Hafez, M.M., Bahnassy, A.A., Hassan, Z.K., Mansour, T., Kamal, M.M., Khaled, H.M., 2008. Genetic profile of Egyptian hepatocellular-carcinoma associated with hepatitis C virus Genotype 4 by 15 K cDNA microarray: preliminary study. BMC Res Notes 1, 106.

## Discussion

Our study aimed at evaluating the effects of time to cryopreservation on both RNA integrity and gene expression variations by analyzing three hepatocellular carcinomas and three lung carcinomas that were snap-frozen at different times after surgical resection.

No deterioration of the RINs was observed with increasing harvest time, even after 120 minutes. This, combined with the quality controls of the arrays, indicates that stress induced by delay to cryopreservation on surgically resected liver and lung tumors had limited impact on both integrity of extracted RNAs and microarray performance. These results corroborate previous observations by Strand *et al*. and Opitz L *et al*. that RIN as low as 5 to 6 is suitable for gene expression measurement and we extend this quality control criterion to genome-wide expression analysis [44], [45]. Interestingly, a recent study suggested that delaying lung tumor tissue harvest for about 30 minutes from surgery has a significant impact on the expression of approximately 25% of the genes [46]. The authors argued that snap-freezing without conservation in *RNA later* had a significant impact on RNA quality and integrity. However, it should be noted that the RIN values of their snap-frozen samples ranged from 3.3 to 5.6, perhaps accounting for the high proportion of apparently deregulated genes. Inappropriate RNA integrity has been reported as a source of bias in gene expression measurements [47], [48].

RNA decay is a tightly controlled and one of the key processes that control the steady-state level of gene expression. Our findings in conjunction with some other studies prove that RNA degradation in lung [19], [49] and liver [50] is limited when samples are maintained at room temperature for up to 2 hours after surgical resection. This observation is also true for most of human tissue types as summarized in Ma *et al*. [14]. Using microarray analysis of RNA samples obtained from mouse embryonic stem cells, Sharova *et al.* have evaluated the rate of mRNA decay for 19,977 non-redundant genes. They found that the median estimated half-life was 7.1 h and that only about 60 genes, including PRDM1, MYC, GADD45G, FOXA2, HES5 and TRIB1, had mRNA with half-lives less than 1 h [51]. However, it cannot be excluded that microarray-based analysis is not appropriate to detect very labile mRNA, thus selecting against their representation in whole gene expression datasets.

Even though RNA degradation within the first hour following surgical resection and before snap-freezing is limited, the process of collection and, specifically the lag time between resection and cryopreservation represent a complex form of metabolic and micro-structural stresses underlying a condition often defined as “warm ischemia”. This complex process may induce significant changes in the level of expression of particular genes. Identification of such changes is an important concern because they may bias the interpretation of transcriptomic data on resected tumor samples. We have attempted to identify a gene expression signature characteristic of the warm-ischemia. Taken together, the time-course analysis of genome-wide data from HCC and LC did not reveal any consistent group of genes, even considering genes involved in inflammatory and immune responses and in cellular growth that were previously reported to be altered during the period between tumor resection and snap-freezing [20]; [52]. When analyzing lung and liver cancer samples separately, no significant deregulated genes were detected over 2 hours harvesting time in LC, consistent with results of Blackhall *et al*. who observed similar gene expression profiles in frozen LC samples regardless of the time between tissue resection and cryopreservation [19].

In contrast to LC, our study in HCC identified 12 genes, representing less than 0.05% of all genes tested, with differential expression in relation to delayed time to freezing. This small number and low proportion of genes is consistent with observations by others in other tissues exposed to warm ischemia [45], [52], [53], [54]. Among these 12 genes, 50% were up-regulated and 50% down-regulated. The *CYP2E* gene, a member of cytochrome P450 family, and the *CLDN2* gene coding for a claudin protein, were significantly induced by warm ischemia at 2 hours post-resection. Of note, Wang *et al* described overexpression of *CYP2E1* in HCC [55] and Maass et al. also found an increased expression of CYP2E exclusively in HCV-induced HCC and also reported over-expression of another member of the claudin family; CLDN10 in HCV-related HCC [56]. Among the 6 down-regulated genes, 4 encoded metallothioneins (MT1E, MT1F, MT1G and MT1H). Down-regulation of metallothioneins has been previously reported in HCC [57], [58], [59] and has been proposed to be associated with defective response to oxidative stress [60]. Specific HCC genes reported in the literature [23], [24], [25], [26], [27], showed highly variable gene expression in our HCC dataset. This raises the possibility that ischemia stress associated with tumor removal may act as potential confounding factor to relevant biological HCC signature. This argument is further supported by the fact that of the 34 genes reported deregulated at least in 4 studies of the Liverome database, we identified one gene MT1F as significantly deregulated under warm ischemia following HCC surgical resection.

Overall, our observations emphasize the importance of identifying tissue-specific genes deregulated following surgical resection, in order to avoid misinterpreting changes in expression induced by warm ischemia as pathologically significant changes.

## Conclusions

Altogether, our findings and previous studies suggest that the effects of warm ischemia induced by the time that elapsed between surgical resection and snap-freezing are minimal within the first two hour post-resection, and that any significant changes in expression induced by warm ischemia are likely to be tissue-specific rather than a systematic expression profiling signature common in all tissue types.

In this study we have only considered the effect of time that elapsed between surgical resection and snap-freezing on RNA integrity and genome-wide expression profiling in two cancer sites, each including only 3 distinct cases. Larger series of different cancer sites, various time points of delay to cryopreservation and several other parameters in tissue handling protocols that may affect RNA integrity (*e.g.,* the addition of a preservative, the repeated freeze-thawing, the sizing and the composition of tissue, the storage container, the speeding of freezing and the final temperature) should be investigated and validated to constitute an integral part of tissue handling recommendations. While recommendations on freezing tissue for expression analysis do exist [17], [61], [62], a precise assessment of the effects of warm ischemia on global gene expression of different cancer sites and tumor types will also help to provide guidelines and recommendations not only for optimal tumor collection and storage but also for optimal interpretation of the gene expression results. The integration of biobanking best practices with gene expression analysis best practices is the key element for biobank to ensure the distribution of high-quality samples and for clinicians and researchers to minimize misinterpretations of global gene expression results.

## Materials and Methods

### 1. Ethical Statement

Tissue specimens were obtained during surgery, according to procedures established by the Tumor Bank of the hospital of Caen, France. Information leaflets were given to the patients regarding use of their biological samples for research. Patients were invited to contact a representative of the tumor bank if they wished to refuse this use. In our series no refusal was recorded. The study was approved by the local ethics committee (Comité de Protection des Personnes Nord Ouest III) on 7^th^ March 2009. This project (IARC reference 09–13) was cleared after ethical review by the IARC (International Agency for Research on Cancer) Institutional Review Board on 29^th^ September 2009. The data were analyzed anonymously.

### 2. Tissue Specimens

This study was conducted on 2 types of tumor, hepatocellular carcinoma (HCC) and lung carcinoma (LC), selected for their incidence, their vascularization and their cellular homogeneity. For each tumor type, three patients were selected (HCC4, HCC5, HCC6, LC2, LC5 and LC6). HCC is the most common liver cancer and surgery is the standard treatment. Poorly to moderately differentiated HCC tumors, with large axis superior or equal to 3 cm, were selected. Lung carcinoma is one of the most frequent cancers in France. Moderately differentiated squamous cell carcinoma tumors, with large axis superior to 3 cm, were selected. Age at diagnosis of patients with HCC was [60–80 years] and [55–76 years] for patients with LC.

Each tumor was processed by a pathologist within the operating theatre directly after resection and divided into several samples measuring approximately 0.125 cm^3^ (0.5×0.5×0.5 cm). For each experimental condition, a sample was extracted from the center and from the periphery of the tumor, and each of these was placed in a cryotube, maintained at room temperature and then frozen in liquid nitrogen at different times: 5 minutes (t5, reference time), 15 minutes (t15), 30 minutes (t30) and 120 minutes (t120). The 48 tumor samples (24 HCC samples from 3 patients and 8 experimental conditions and 24 LC samples from 3 patients and the same 8 experimental conditions) were then stored at −80°C until extraction and analysis. The details of samples and experimental conditions are summarized in Table 1.

### 3. RNA Isolation

For each sample, RNA extraction was performed using the NucleoSpin RNA II kit (Macherey-Nagel) according to the manufacturer’s instructions. RNA concentration and RNA purity were evaluated with the Nanodrop® (Thermo Scientific). RNA integrity and quantification were characterized by measuring the 28 s/18 s rRNA ratio and RIN (RNA Integrity Number) using the Agilent 2100 bioanalyzer instrument and the RNA 6000 Nano kit. The RIN software classifies the integrity of eukaryotic total RNAs on a scale of 1 to 10, from most to least degraded.

### 4. Whole Genome Expression Profiling of Frozen Tissues

Genome-wide gene expression profiling analysis was performed on Illumina HumanHT-12 v3 Expression BeadChips, providing a coverage of more than 24,000 annotated genes (48,783 probes corresponding to 1 to 3 probes per gene) including well characterized genes and splice variants. Candidate probe sequences included on the HumanHT-12 v3 Expression BeadChip derive from the National Center for Biotechnology Information Reference Sequence (NCBI) RefSeq (Build 36.2, Rel 22) and the UniGene (Build 199) databases. Using the Illumina TotalPrep RNA Amplification Kit (Ambion®), 500 ng of extracted RNAs were converted to cDNAs and subsequent biotin labeled single-stranded cRNAs. The distribution of homogeneous *in vitro* transcription products (cRNAs) was checked with the Agilent 2100 bioanalyzer instrument and the RNA 6000 Nano kit. 750 ng of biotin labeled cRNAs of the 48 samples were hybridized overnight to 4 HumanHT-12 Expression BeadChips. Subsequent steps included washing, streptavadin-Cy3 staining and scanning of the arrays on an Illumina BeadArray Reader. Fluorescence emission by Cy3 was quantitatively detected for downstream analysis. The Illumina Genome Studio V2010.2 was used to obtain the signal values (AVG-Signal), with no normalization and no background subtraction. Data quality controls were performed using internal controls present on the HumanHT-12 beadchip and were visualized as a control summary plot. The microarray experiments are MIAME (Minimum Information About a Microarray Experiment) compliant and have been deposited at the NCBI Gene Expression Omnibus (GEO) database (http://www.ncbi.nlm.nih.gov/geo) under accession GSE41160.

### 5. Statistical Analysis

#### 5.1. Quality control and preliminary analysis

RNA quality was tested for association with time to cryopreservation in two ways, before the data were normalized. First, the continuous RIN values were tested for association with time by linear regression. Second, the number of genes detected in each sample was analyzed by Poisson regression. Detection of an RNA by a probe was defined by significantly (p<0.01) higher intensity than both the gene- and sample-specific mean of negative control probes. Array and array position were included as random effects, patient ID, center versus peripheral source, RIN and tumor type as fixed effects in each of these regressions.

The ratio of centiles P95/P05 calculated for each sample prior to normalisation reflects the overall strength of the signal compared to the background. We considered ratios above 10 to be acceptable.

Bead-set standard deviations were observed to be approximately proportional to mean expression levels for each probe, suggesting the data should be log-transformed. The log-transformed data appeared to be homoskedastic.

For each sample, scatter plots were generated to compare the t5 tissue to the corresponding tissue frozen after delay (t15, t30 and t120), using the log scale.

We also calculated the Pearson correlation coefficient between log-expression levels of the central and peripheral samples, for each tumor and each time to cryopreservation.

#### 5.2. Overall expression changes

We first tested for a trend in expression over all genes. The transformed expression levels were averaged with inverse-variance weighting to obtain a minimum variance estimate of the mean log expression level (log of the geometric mean) over all probes.

The data were modelled via a random-intercept linear mixed regression model, with over-all rates of change estimated as percent-change per hour. To attempt to detect any non-linear behaviour, a term quadratic in time was tested. The data were also stratified by tumor type, to examine if there were detectable differences between tumor sites, and by peripheral versus central origin of the sample. In order to investigate whether expression changes were more pronounced in genes with higher or lower levels of expression, the above analyses were repeated after restricting to those genes in the highest or lowest 5% of geometric mean expression across all time points.

#### 5.3. Individual genes

Regression analysis of time course expression data for individual genes was performed using BRB-ArrayTools software v4.2 developed by Dr Richard Simon and BRB-ArrayTools Development Team. Data were log-transformed and quantile normalized without background subtraction as described above, but with the exclusion of any probe showing excess dispersion (defined by more than 80% of individual probe values differing from the median by more than 1.5-fold). The BRB-ArrayTools time course analysis model fits the same quadratic model as used over-all, with null hypothesis that both linear and quadratic terms were zero. Genes for which this hypothesis was rejected were identified. The tests were performed at a false discovery rate (FDR) threshold of 0.05 [18].

#### 5.4 Exploratory Cluster analysis

Unsupervised hierarchical clustering of samples was performed using both Genome Studio V2010.2 and BRB-ArrayTools software v4.2.

#### 5.5. Analysis restricted to pre-specified ischemic genes and HCC-specific genes

Twenty-one genes that have been previously reported as deregulated at an early stage after surgery were specifically selected for further analysis in the experimental dataset [14], [19], [20]. This includes *JNK3, JUNB, AP1B1, AP1S1, AP1M1, AP1S1, CA-IX, HHR6B, PRSS25, FOS, HIF1A, HO-1, JUND, JUN, KRT19, KRT20, CEA, KLF6, MDM4, FBLN2, FGRF4.* Overall rates of expression changes were analyzed as described in the overall changes in expression paragraph, but since these probes may potentially be distinguished by either increasing or decreasing expression with time, a further two-stage analysis was carried out. A quadratic polynomial time effect was fitted separately for each probe. From each of these, the estimated coefficients were compared with the distribution of the values from the entire set of approximately 48,800 probes, in order to observe if they tended to the extremes of this distribution. This was tested using Neyman’s method of smooth contrasts with a quadratic contrast [21] The method was first applied to the ranks of rates of change as estimated, and to the ranks of rates of change after normalizing by the standard errors of these estimates. The normalization step was performed because extreme values in a distribution are expected be more influenced by random noise, and the normalization reduces this effect. The Neyman contrast tests were performed on the complete set of samples, then restricted to the HCC samples and the LC samples.

Finally, in order to examine whether some reported biologically significant HCC genes could also be found deregulated in a context of warm ischemia associated with tumor freezing delay, we restricted our analysis of the rates of expression changes in 34 HCC genes reported to be deregulated in more than 4 studies in the public Liverome database [22]. The list of the 34 selected genes is provided in Table 3. The same Neyman contrast tests were applied. In addition, to determine if there was consistency of direction of effect, we compared the rate of change (slope) estimated in our data with the directions reported in the literature. Multiple probes in the same gene were averaged with inverse-variance weighting. Since many of the genes were identified in multiple publications but not always in the same direction, we used block-logistic regression to compare the slope with the proportion of reports of up-regulation among those reporting either up or down regulation.
